# Rapid diagnosis of COVID-19 using FT-IR ATR spectroscopy and machine learning

**DOI:** 10.1038/s41598-021-93511-2

**Published:** 2021-10-11

**Authors:** Marcelo Saito Nogueira, Leonardo Barbosa Leal, Wena Dantas Marcarini, Raquel Lemos Pimentel, Matheus Muller, Paula Frizera Vassallo, Luciene Cristina Gastalho Campos, Leonardo dos Santos, Wilson Barros Luiz, José Geraldo Mill, Valerio Garrone Barauna, Luis Felipe das Chagas e Silva de Carvalho

**Affiliations:** 1 Tyndall National Institute, University College Cork, Lee Maltings Complex, Dyke Parade, Cork, T12R5CP Ireland; 2 Department of Physiological Sciences, Federal University of Espírito Santo (UFES), Vitória, Brazil; 3 Universidade de Taubaté, Taubaté, Brazil; 4 Centro Universitário Braz Cubas, Mogi das Cruzes, Brazil; 5 Clinical Hospital, Federal University of Minas Gerais, Belo Horizonte, MG Brazil; 6 Department of Biological Science, Santa Cruz State University, Ilhéus, BA Brazil; 7 Faculdade Vale do Cricaré, São Matheus, Brazil

**Keywords:** Translational research, Applied optics, Biophotonics, Viral infection

## Abstract

Early diagnosis of COVID-19 in suspected patients is essential for contagion control and damage reduction strategies. We investigated the applicability of attenuated total reflection (ATR) Fourier transform infrared (FTIR) spectroscopy associated with machine learning in oropharyngeal swab suspension fluid to predict COVID-19 positive samples. The study included samples of 243 patients from two Brazilian States. Samples were transported by using different viral transport mediums (liquid 1 or 2). Clinical COVID-19 diagnosis was performed by the RT-PCR. We built a classification model based on partial least squares (PLS) associated with cosine k-nearest neighbours (KNN). Our analysis led to 84% and 87% sensitivity, 66% and 64% specificity, and 76.9% and 78.4% accuracy for samples of liquids 1 and 2, respectively. Based on this proof-of-concept study, we believe this method could offer a simple, label-free, cost-effective solution for high-throughput screening of suspect patients for COVID-19 in health care centres and emergency departments.

## Introduction

In early 2020, a new strain of coronavirus called SARS-CoV-2 became a major health problem worldwide. After an epidemic outbreak in Wuhan-China in late 2019, it quickly became a pandemic with serious consequences to the healthcare system and also at social, political and economic sectors worldwide^1–4^. Although research is currently being carried out to investigate the biomolecular mechanisms of virus spreading, no effective treatment^5,6^ and vaccine^7^ has been developed yet. In addition, prescribing diagnostic assays for patients with clear symptoms has not been sufficient to contain the COVID-19 transmission rate^8^. Therefore, an increase in the number of cases is still expected in many countries around the world.

In this pandemic, extensive testing of the asymptomatic population and early detection of COVID-19 in suspected patients are crucial for contagion control and damage reduction strategies. However, due to technical issues, current tests are complex and costly. Although real‐time reverse‐transcriptase polymerase chain reaction (RT-PCR) is actually the gold standard tests for COVID-19, usually performed with a sample from the nasopharyngeal swab, the current data indicate that it is not very sensitive due to fluctuation of viral load, which significantly further reduces after the 10th day of symptoms^9^. Thus, health care systems around the world have used rapid serological tests indicating past exposure to SARS-CoV-2, which also show varied efficacy, and similar to RT-PCR, have shown a percentage of false negatives^9^. Thus, none of the tests currently available has completely satisfactory performance, and the search for rapid and low-cost tests with adequate sensitivity is critical.

In addition to social isolation, governments are looking to rapidly expand testing capabilities as the major means to battle the COVID-19 pandemic. Serology testing is mainly used for surveillance and epidemiological purposes, since it only checks for past exposition to the virus. It was demonstrated that immune response to the new coronavirus takes 1–2 weeks to occur^10^, which justifies why serological tests are not appropriate for detection in the acute phase of the disease. Actually, RT‐PCR assay has been widely used as the gold standard to detect SARS‐CoV‐2 in respiratory samples such as nasopharyngeal swabs or bronchial aspirate and, thus, to indicate isolation and treatment, discharge, or transfer to units for patients diagnosed with COVID-19. In the present study we used symptomatic patients tested by RT-PCR for definition of confirmed cases (positive) or not-a-case (negative) by the first testing.

Although the in vitro sensitivity of RT-PCR tests is high, the sensitivity of the nasopharyngeal RT-PCR swab tests for diagnosing COVID-19 in clinical settings is questionable. It is well known that the accuracy of the test depends not only on its intrinsic characteristics and the time-window of viral replication, but also on the selection of the population to be tested. In a recent pre-proof meta-analysis of the accuracy of diagnostic tests for COVID-19, a number of studies show false negatives by RT-PCR if the viral load is insufficient or if the time-window of viral replication is inadequate^9^. This systematic review evidenced an averaged sensitivity of 73.3% (95% CI 68.1–78.0%) nasopharyngeal/throat swabs on data collected from seven clinical trials. The selection of the tested population interferes with the accuracy of the test, and an even worse sensitivity (62%) has been reported for mild cases^11^.

Thus, due to the inherent characteristics of tests currently approved by regulatory agencies, there is a need for the association of different tests, collection of multiple samples, collections in different regions and at repeated time-points, in order to obtain a definitive diagnosis of COVID-19, which makes the pandemic containment even more complex and costly. In this context, the combination of different diagnostic tests is highly useful to achieve adequate sensitivity and specificity^9^.

Attenuated total reflection Fourier transform infrared (ATR-FTIR) spectroscopy associated with machine learning methods could be a potential alternative method for diagnosis of COVID-19, as it is simple, label-free and cost-effective. This technique has shown promise as a diagnostic or screening tool in several diseases such as cancer^12–14^, diabetes, hypertension, and physiological stress^15,16^. In 2018, Leal et al.^15^ and Baker et al.^17^ pointed out that small samples of biofluids (saliva, blood and urine) could be effective for the diagnosis of a wide range of diseases, including infectious diseases^18^. The application of vibrational optical techniques for COVID-19 detection was discussed by later papers, especially in the context of enabling high-throughput screening and combination with telemedicine to contain the virus spreading^19,20^. Within this context, the objective of the present study is to show that ATR-FTIR spectroscopy associated with machine learning methods for analysis of swab suspension fluids from suspected patients is capable of becoming a novel real-time, cost-effective diagnostic tool for COVID-19.

## Results and discussion

For LIQUID 1, a total of 65 suspected patients went through COVID-19 screening and of them 40 were cases confirmed (positive) by RT-PCR and 25 were not-a-case (negative); while from 178 suspected patients screened for LIQUID 2, 111 patients were confirmed as positive and 67 patients as negative. The group of patients from LIQUID 1, presents the age (mean ± SD) of 46.2 ± 15.9 and 64.7% were women, with 63.9% positives and 36.1% negatives, and for LIQUID 2 the age was 50.9 ± 18.2, 64% were women, with 59.1% positives and 40.9% negatives.

### LIQUID 1 classification model

**Figure 1:**
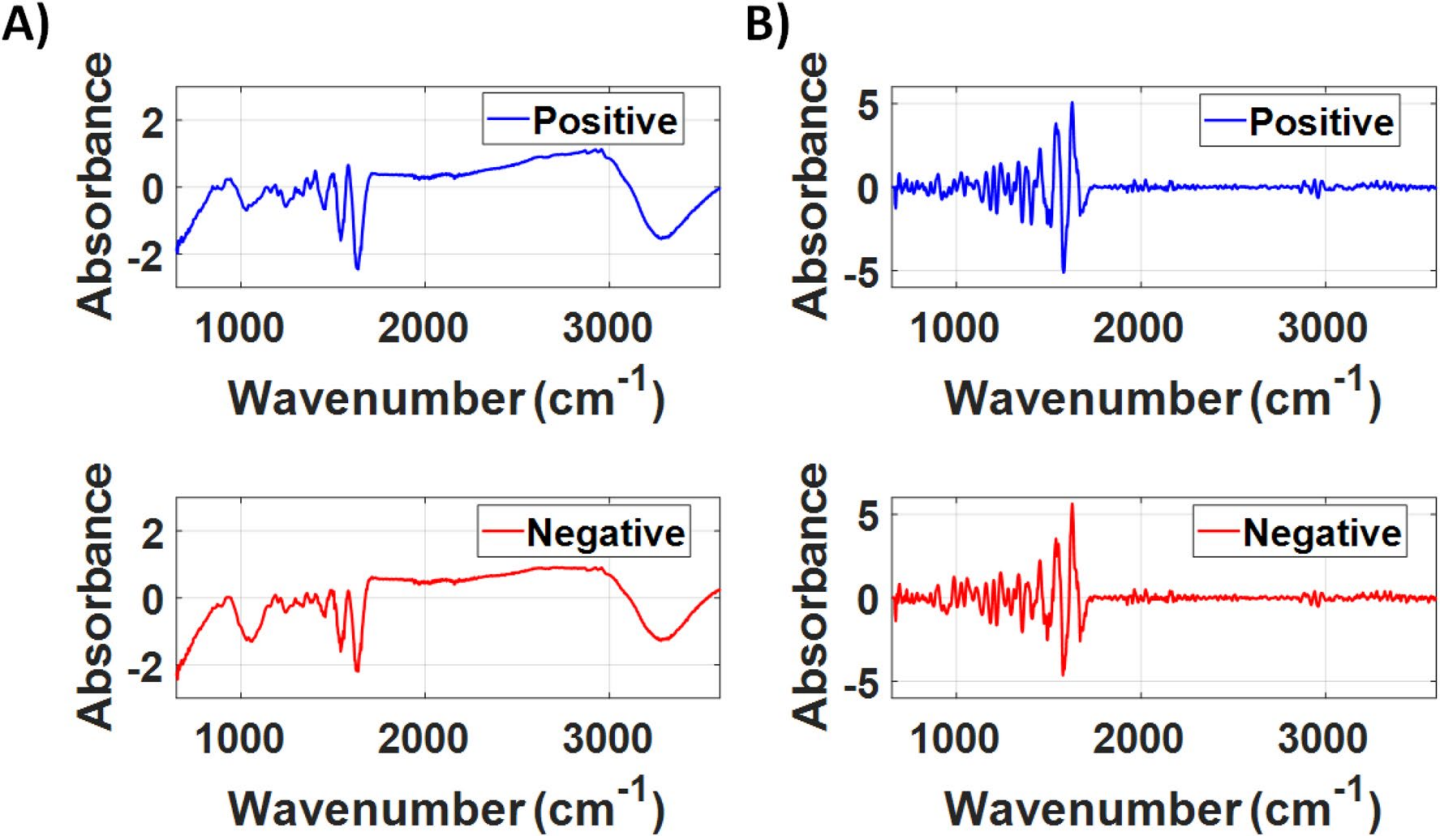
(**A**) Averaged FTIR spectra and (**B**) averaged second derivative of the FTIR spectra of the COVID-19 positive and negative groups of the LIQUID 1 dataset.

Figure 1 shows the average spectra after the subtraction of the spectrum of LIQUID 1 and the second derivative of the COVID-19 positive and COVID-19 negative groups for the LIQUID 1 dataset. The second derivative removes the background present in the raw ATR-FTIR spectra to enhance the visualization of the features of COVID-19 positive and negative spectra.

**Figure 2:**
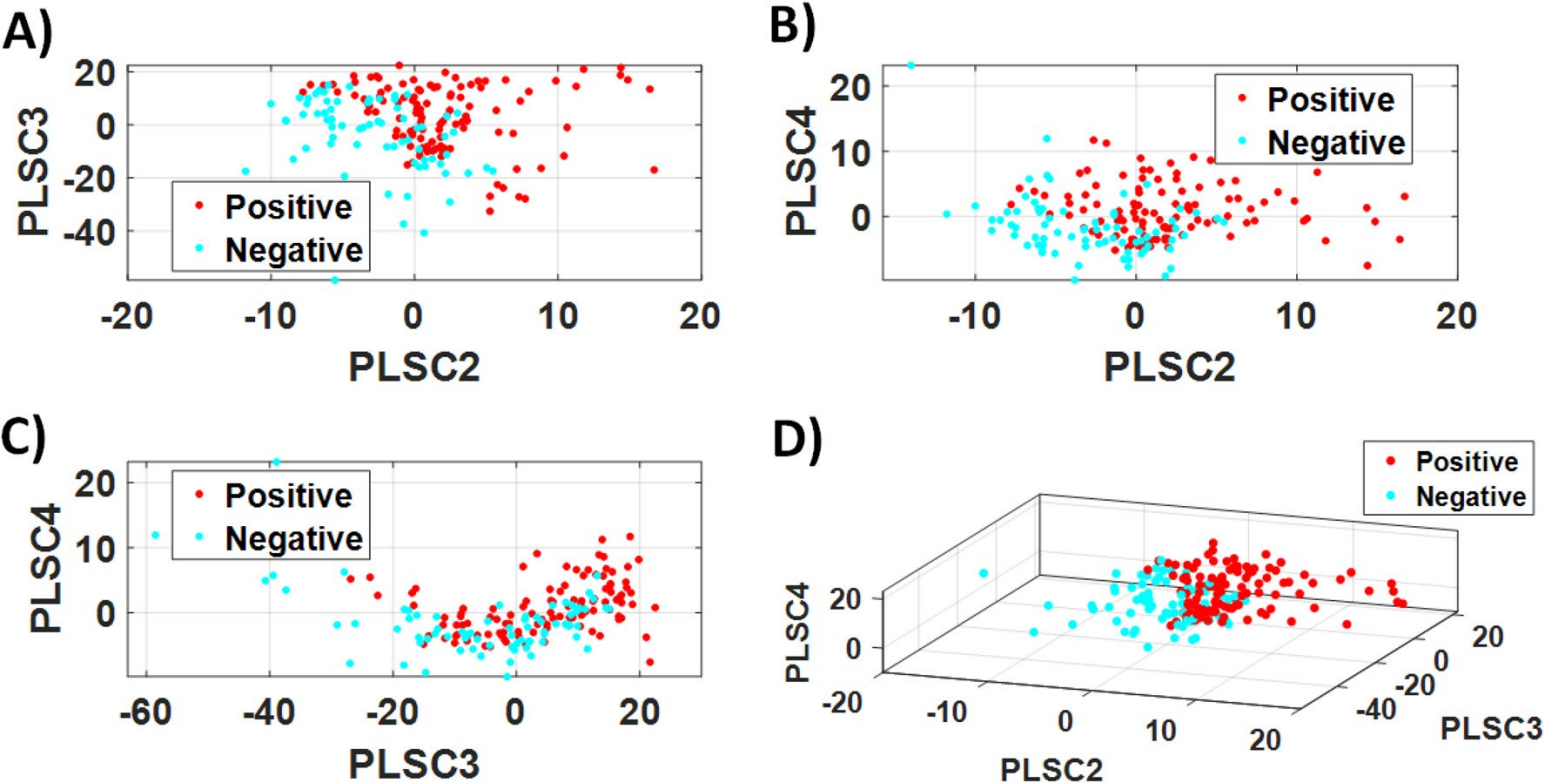
PLS score plots of (**A**) PLSC2 × PLSC3, (**B**) PLSC2 × PLSC4, (**C**) PLSC3 × PLSC4, (**D**) PLSC2 × PLSC3 × PLSC4 for COVID-19 positive and negative groups of the LIQUID 1 dataset.

The second derivative of the FTIR spectra were used to calculate the PLS components (PLSC) for differentiation of the COVID-19 positive and negative groups of the LIQUID 1 dataset. The differentiation between these groups is illustrated in Fig. 2, which shows the PLS scores for combinations of PLSC2, PLSC3 and PLSC4. Most of the differentiation could be observed for combinations including PLSC2.

**Figure 3:**
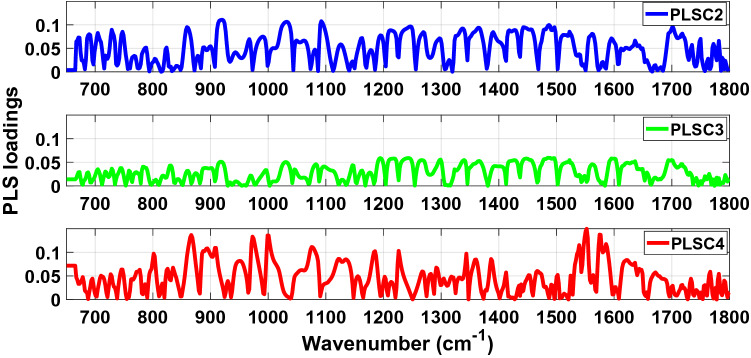
Absolute values of PLS loadings of PLSC2 (blue), PLSC3 (green) and PLSC4 (red) in the fingerprint spectral region between 650–1800 cm^−1^ for our analysis of the LIQUID 1 dataset.

**Figure 4:**
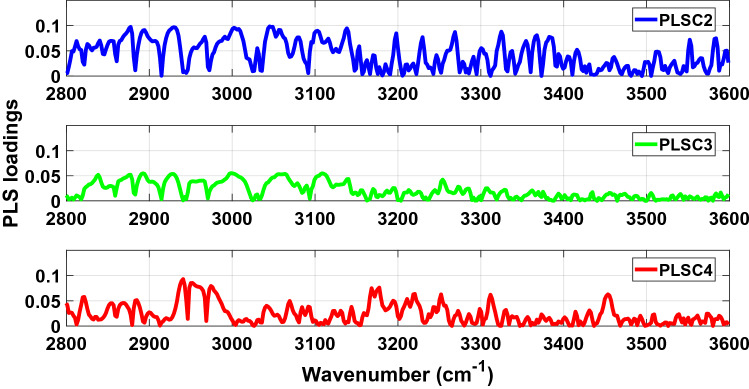
Absolute values of PLS loadings of PLSC2 (blue), PLSC3 (green) and PLSC4 (red) in the high wavenumber spectral region between 2800–3600 cm^−1^ for our analysis of the LIQUID 1 dataset.

The absolute values of PLS loadings of PLSC2, PLSC3 and PLSC4 used to determine the main vibrational modes and biochemical components (Table 1) associated with the discrimination between COVID-19 positive and negative groups are illustrated in Fig. 3 (fingerprint region) and Fig. 4 (high wavenumber region). Peaks can be found over a wide range of wavenumbers. Most of the narrow peaks were observed between 665–760 cm^−1^, 1030–1250 cm^−1^, 1725–1800 cm^−1^, and 3130–3600 cm^−1^.

**Table 1:** Main vibrational modes present in the fingerprint region between 650–1800 cm^−1^ and the high wavenumber region between 2800–3000 cm^−1^ of the PLS loading spectra of PLSC2, PLSC3 and PLSC4 for the analysis of the LIQUID 1 dataset (according to Movasaghi et al.^21^ and Naseer et al.^22^).

	Wavenumber (cm^−1^)	Vibrational mode	Structural component
PLSC2	921 (924)	?	Membrane lipids (phospholipids)
	1092	Stretching PO2 2 symmetric (phosphate II) Nasym(C–O–C)	(polysaccharides-cellulose)
	2878 (2880)	Asymmetric stretch of –CH2	Methylene
PLSC3	1299	Deformation N–H	Cytosine
	1146	C–O bond	Phosphate and oligosaccharides
	2838	Stretching C–H	Methoxy
PLSC4	1552 (1550)	CN stretch and NH bend	Amide I
	867	?	Left-handed helix DNA (Z form)
	2941	?	?

The interpretation of the coronavirus samples detected in patients with COVID-19 was performed by associating vibrational modes derived from spectral data with sample biochemical/structural components. The peak at 921 cm^−1^ is not related to the vibrational modes and the structural components of the Table 1^21^. Peak frequency 1092 is related to the vibrational modes stretching PO2 2 symmetric (phosphate II) (50) nasym (C–O–C) and the cellulose polysaccharide structural component that stimulates the production of saliva. The 1299 (94/95/96) cm^−1^ peak represents the vibrational mode of the deformation N–H and the cytosine structure, which is suggested to be involved in hypercytokinemia in severe cases of COVID-19^8,23–25^.

The sharp increase in these proteins generates a hyperinflammatory response that leads to organ dysfunction and, in several cases, multiple organ failure. In the band at 1146 cm^−1^, phosphates are also used as modular blocks of various substances, including those used by the cell for energy, cell membranes and DNA^26,27^, and can be indicated for the treatment of flu-like symptoms; oligosaccharides are found on the outer surface of the plasma membrane, in the blood, in the cellular matrix and in most of the secreted proteins^8,23–25^.

The peak at 2838 cm^−1^, the structural mode found was stretching C–H^21^, and the structural component methoxy can be related to esters and can be used in medicine, as an anesthetic and in the preparation of medicines^8,23–25^. Bands at 1552 cm^−1^ and 867 cm^−1^ were not related to vibrational modes, but to structural components, such as the base ring and left-handed helix DNA (Z form)^21^.

Recently, ATR-FTIR has already been investigated as a screening/diagnostic tool in medicine. In 2019, the use of this technique was reported in the screening of patients with brain cancer, achieving sensitivity of 93.2% and specificity of 92.8% in the identification of high-risk patients indicated for definitive diagnostic tests (more expensive), thus saving time and cost^28^. Moreover, vibrational spectroscopy has been used with very good results in different areas of health science, as brain cancer^28^, oral cancer^29–31^ and prostate cancer^32^. In infectious diseases, a similar study was done to discriminate patients with Human immunodeficiency virus (HIV) infection by ATR-FTIR also associated with linear discriminant analysis (LDA) in plasma samples. Interestingly, this analysis proved to be a possible strategy for discrimination against different spectra of HIV infection and co-infection with the hepatitis C virus (AIDS, HIV + HCV or AIDS + HCV)^18^.

**Figure 5:**
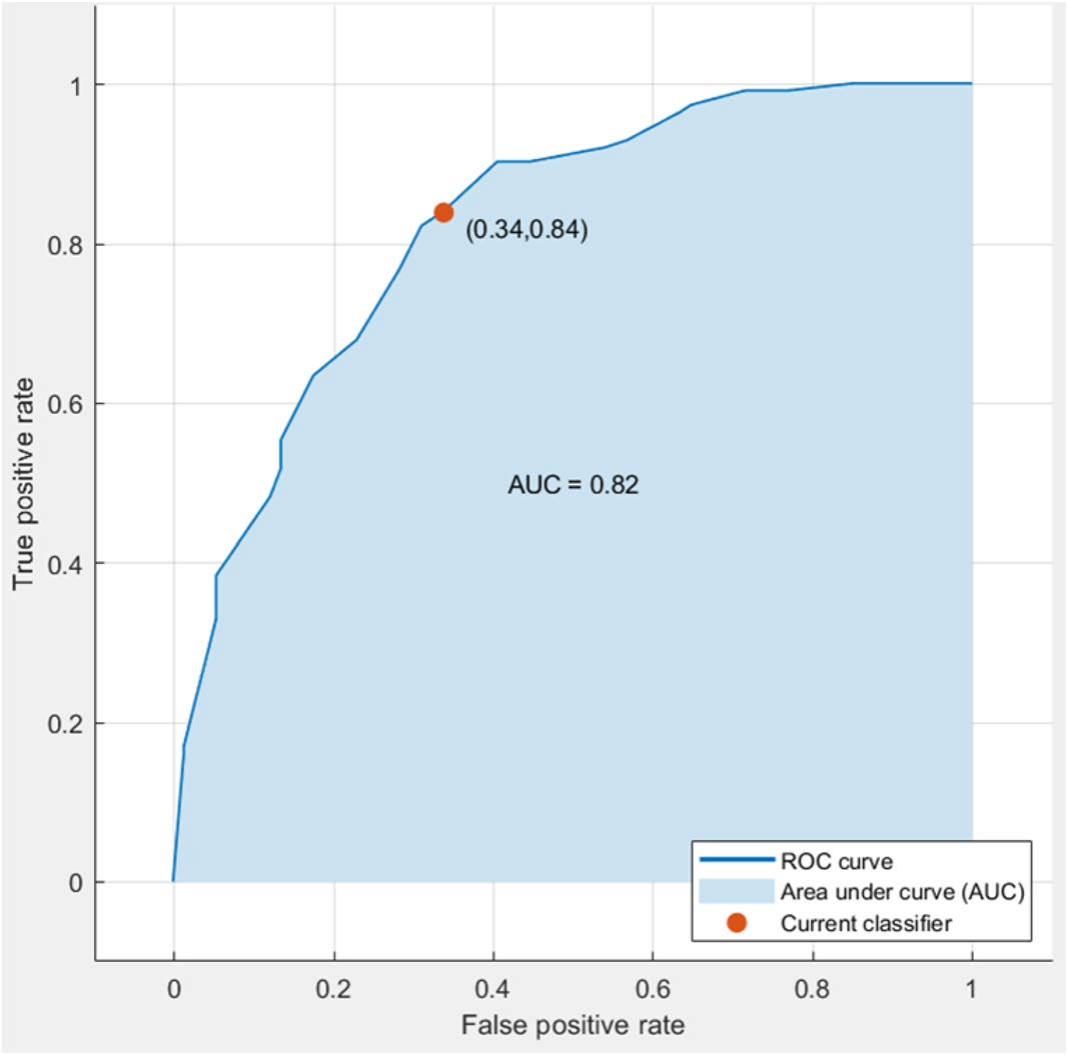
ROC curve suggests that the specificity and sensitivity of best classifier (PLS-cosine KNN) found difference between positive and negative samples for COVID-19 (LIQUID 1 dataset) upon fivefold cross-validation.

In the present study, the multivariate statistical analysis using a PLS-cosine KNN model achieved 84% of sensitivity and 66% of specificity, and 76.9% accuracy upon fivefold cross-validation. Also, the area under the ROC curve (AUC) was 0.82 which is a satisfactory value for a proposed real-time COVID-19 detection method (Fig. 5).

### LIQUID 2 classification model

**Figure 6:**
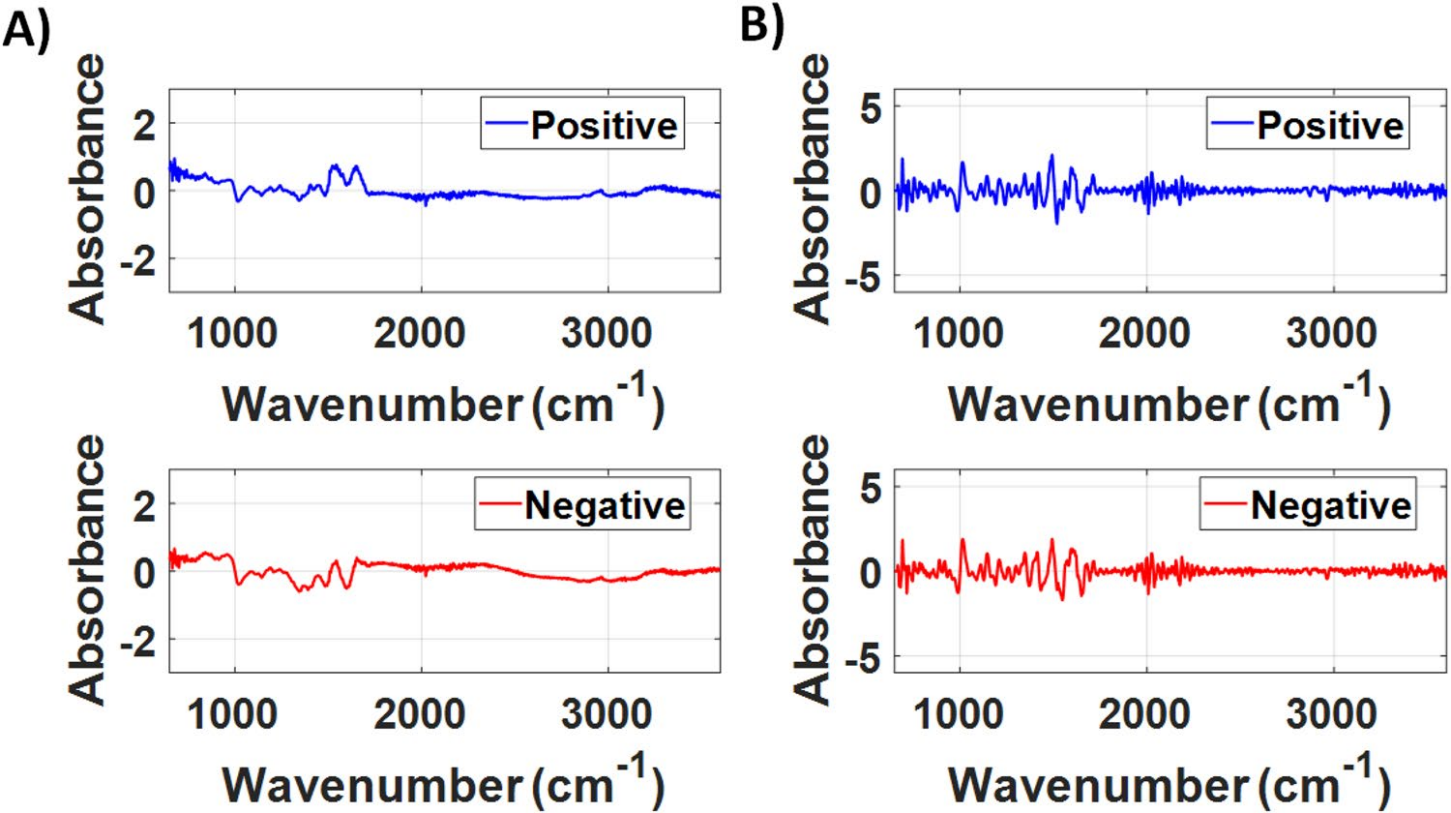
(**A**) Averaged FTIR spectra and (**B**) averaged second derivative of the FTIR spectra (**B**) of the COVID-19 positive and negative groups of the LIQUID 2 dataset.

Figure 6 shows the average spectra after the subtraction of the spectrum of LIQUID 2 and the second derivative of the groups COVID-19 positive and COVID-19 negative for the LIQUID 2 dataset. Even though the average spectra are slightly different in shape compared to the LIQUID 2 dataset, which may be due to the different viral transport medium of the swab suspension fluid used in these two locations, the spectral shape of are similar between datasets and differences are evidenced by computing the second derivative of the FTIR spectra.

**Figure 7:**
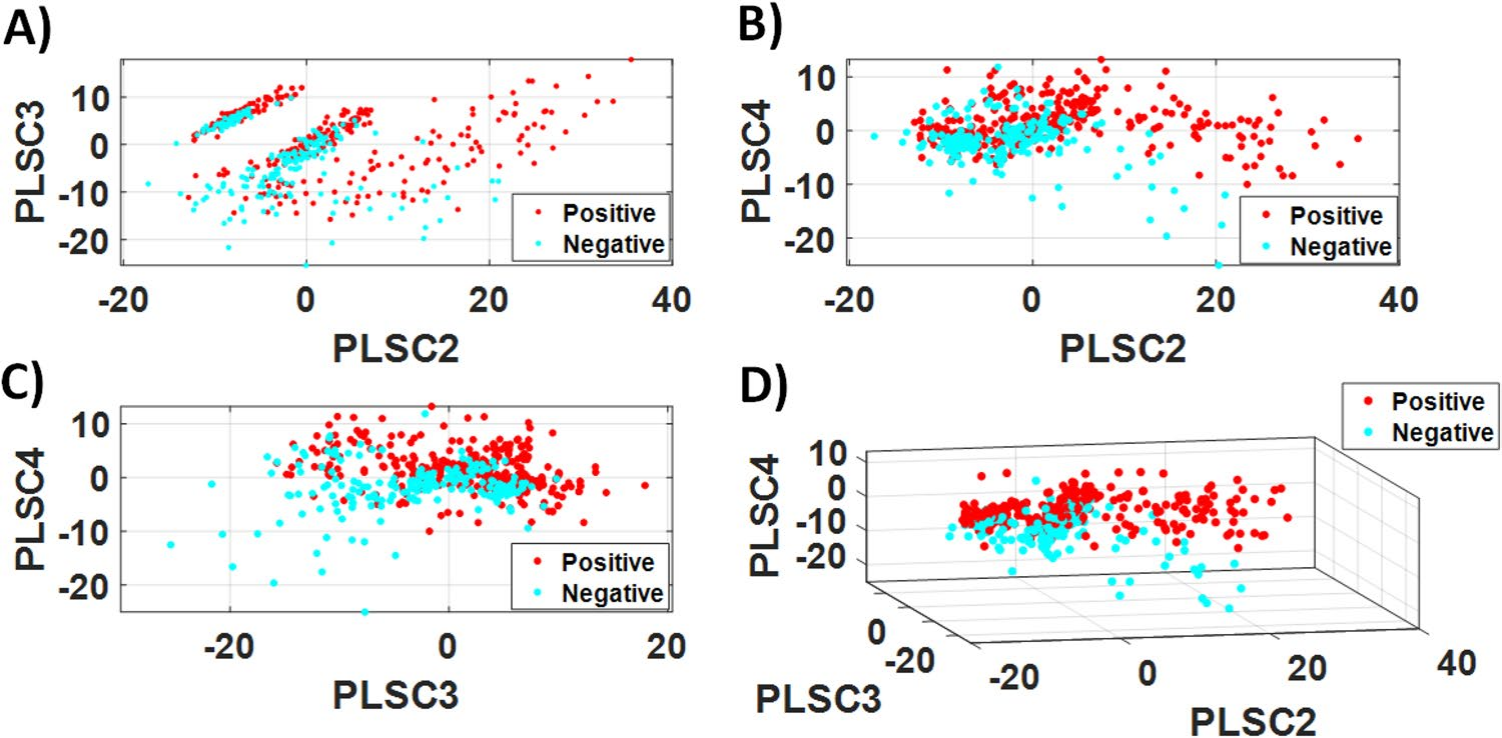
PLS score plots of (**A**) PLSC2 × PLSC3, (**B**) PLSC2 × PLSC4, (**C**) PLSC3 × PLSC4, (**D**) PLSC2 × PLSC3 × PLSC4 for COVID-19 positive and negative groups of the LIQUID 2 dataset.

The PLS score differentiation between COVID-19 positive and negative groups of the LIQUID 2 dataset is can be observed in Fig. 7. In contrast with the analysis of LIQUID 1 dataset, PLSC2 seems to contribute less to the discrimination between groups. Still, combining scores of PLSC2, PLSC3 and PLSC4 could lead to a reasonably good discrimination (Fig. 7D) which can only be confirmed and automated by using the KNN model discussed later in this section.

**Figure 8:**
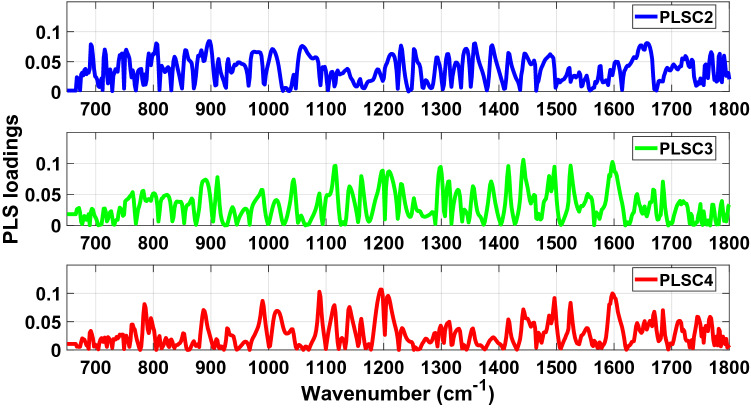
Absolute values of PLS loadings of PLSC2 (blue), PLSC3 (green) and PLSC4 (red) in the fingerprint spectral region between 650–1800 cm^−1^ for our analysis of the LIQUID 2 dataset.

**Figure 9:**
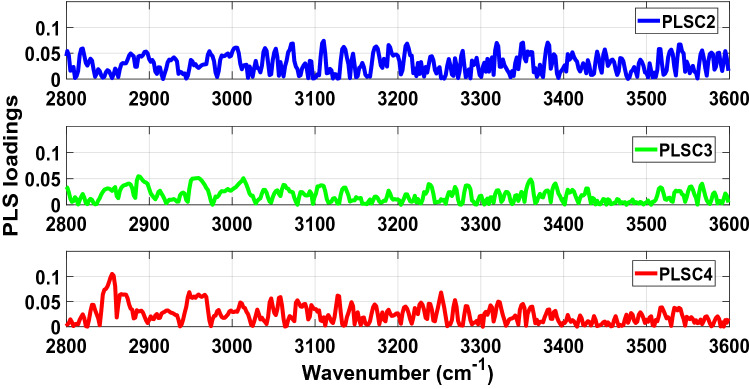
Absolute values of PLS loadings of PLSC2 (blue), PLSC3 (green) and PLSC4 (red) in the high wavenumber spectral region between 2800–3600 cm^−1^ for our analysis of the LIQUID 2 dataset.

In the same way as data was analysed for LIQUID 1 samples, the absolute values of PLS loadings of PLSC2, PLSC3 and PLSC4 (Figs. 8 and 9) were used to understand the sample biochemistry (Table 2) related to differentiation of COVID-19 positive and negative groups. Most of the narrow peaks which could be associated with vibrational modes occurred between 665–780 cm^−1^, 1070–1540 cm^−1^, 1730–1800 cm^−1^, 2800–2845 cm^−1^ and 3020–3600 cm^−1^.

**Table 2:** Main vibrational modes present in the fingerprint region between 650–1800 cm^−1^ and the high wavenumber region between 2800–3000 cm^−1^ of the PLS loading spectra of PLSC2, PLSC3 and PLSC4 for the analysis of the LIQUID 1 dataset (according to Movasaghi et al.^21^ and Naseer et al.^22^).

	Wavenumber (cm^−1^)	Vibrational mode	Structural component
PLSC2	896	?	?
	1359 (58)	Stretching C–O, deformation C–H, deformation N–H	?
	2820	–CH2 and –CH3	Lipids
PLSC3	1442	d(CH2)	Lipids, fatty acids (polysaccharides, pectin)
	1524	vCN, vCC proteins, tyrosine	Amide II
	2913	?	?
PLSC4	1088	Stretching PO2 2 symmetric vibration) in B-form DNA	Phosphate I
	1497	C=C, deformation C–H	Proteins
	2855	Asymmetric CH2 stretching mode of the methylene chains in membrane lipids	Lipids

It can be seen that the 896 cm^−1^ peak is not related to the vibrational modes and the structural components found in the tabulated data from Movasaghi et al.^21^. The peak at wavenumber 1359 (58) cm^−1^, is related to the vibrational modes Stretching C–O, deformation C–H, deformation N–H, but structural components were not found. At the maximum 2820 it has been related to the vibrational mode Stretching N–H (NH3). However, the structural components were not Movasaghi et. al.^21^ table.

Bands at 1442 (44) cm^−1^ are related to the vibrational mode d (CH2), and the structure containing lipids, fatty acids (polysaccharides, pectin). Scientific studies show that fatty acids can alter the lipid composition of cell membranes, which results reduced inflammation due to the production of molecules that are less inflammatory compared to those produced when omega-3 is not present^8,23–25^. At the 1524 cm^−1^ peak, the vibrational mode Stretching C=N, C=C belongs to, but no structural components were found. The peak at 2913 cm^−1^ is not related to vibrational modes and structural components of samples involved in this research.

Through the research, the 1088 cm^−1^ band can be associated with the vibrational modes stretching PO2 2 symmetric vibration found in B-form DNA^26,27^ and the structural component phosphate I^21^, which is involved in processes of energy production inside the cell. The 1497 cm^−1^ band could be related to the vibrational mode of C=C and deformation C–. Finally, the peak found at 2855 (53) cm^−1^ corresponds to the vibrational modes Asymmetric CH2 stretching mode of the methylene chains in membrane lipids and the structural component lipids^21^, which could be related to the composition of the plasma membrane of cells^8,23–25^.

**Figure 10:**
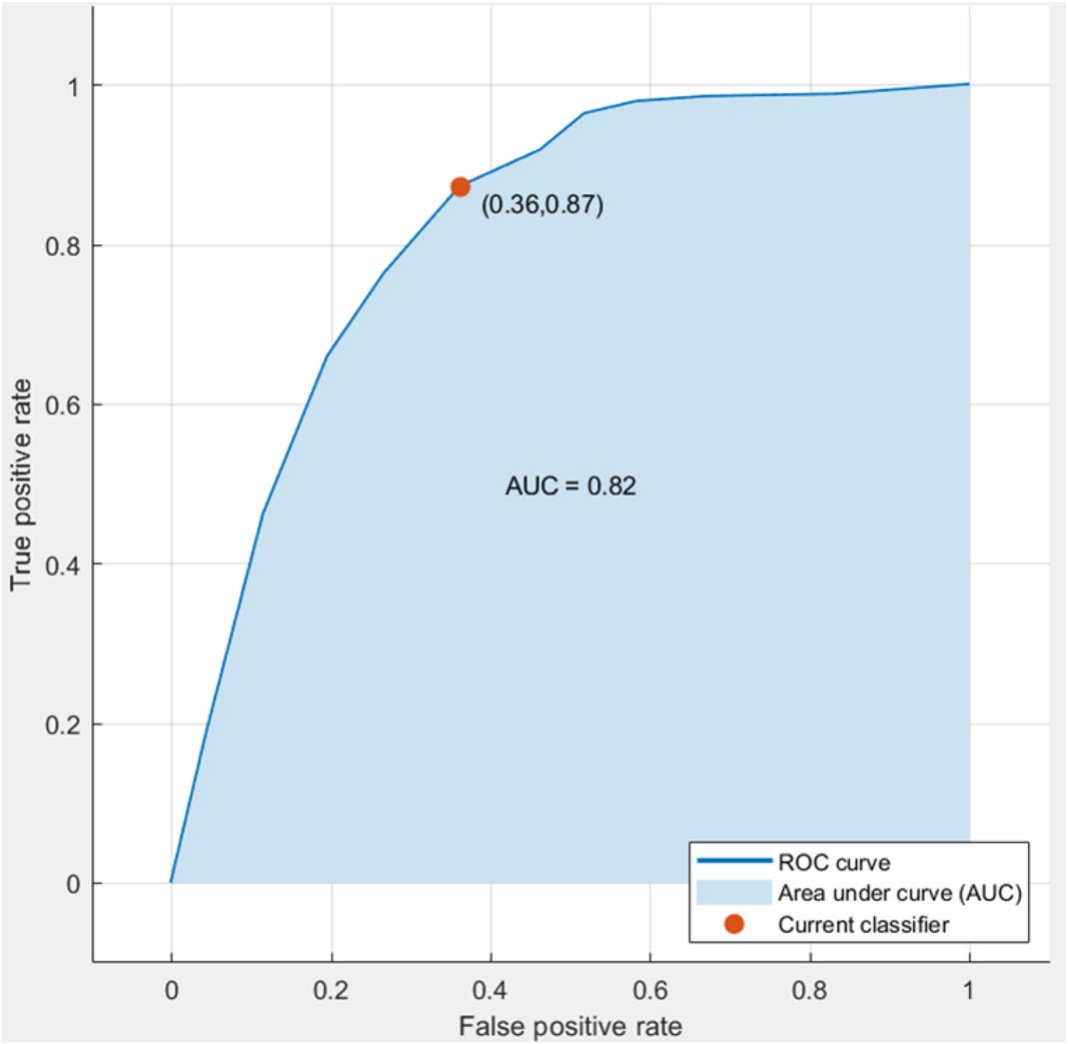
ROC curve suggests that the specificity and sensitivity of the best classifier (PLS-cosine KNN) found difference between positive and negative samples for COVID-19 (LIQUID 2 dataset) upon fivefold cross-validation.

The statistical analysis using a PLS-cosine KNN model on the LIQUID 2 dataset achieved 87% of sensitivity and 64% of specificity, and 78.4% accuracy upon fivefold cross-validation; and the area under the ROC curve (AUC) was 0.82 (Fig. 10), which configure a satisfactory performance for a proposed real-time COVID-19 detection method.

In general, research involving the diagnosis of COVID-19 is considerably difficult, mainly due to the novelty that the disease alone brings. Thus, we are currently working with totally incomplete information regarding the pathophysiology of the disease, even having as much clinical and epidemiological information as possible. Furthermore, the use of the FTIR spectroscopy technique, although it makes the diagnostic challenge even greater, is totally relevant and the research involved is timely, as highlighted by Carvalho and Saito Nogueira in a letter to the editor^19^.

### Current status, limitations and future perspectives

In symptomatic patients presenting dyspnea and low oxygen saturation, imaging tests such as chest X-ray or computed tomography (CT) can help with the clinical diagnosis the COVID-19. Since the CT scan process is fast and relatively simple and CT equipment is adopted worldwide, it enables relatively rapid screening of suspected patients, assessing the severity of the disease, response to treatment, or presence of complications and differential diagnosis^33,34^. However, it is important to note that CT has helped the diagnosis of severe and symptomatic COVID-19 cases, whereas asymptomatic patients do not benefit from this technique, as it is not cost-effective for screening of patients without pulmonary manifestations. Also, CT equipment is not always located in close distances from COVID-19 and at times patients with severe symptoms cannot be transported to centres with the CT equipment.

Yet, clinical experience has demonstrated a poor diagnostic accuracy of chest CT in screening patients with suspected COVID-19 without chest discomfort, difficulty in breathing or pneumonia. In this scenario, all suspect cases still waiting for RT-PCR results, being followed exclusively by its clinical condition to the moment.

Thus, by using our approach with an accurate prediction of the biomolecular test, the decision making could already be done in advance (isolation, treatment or transfer to another health centre or intensive care units, etc.).

It is important to note that FTIR could be a point-of-care, fast-running, low-cost, non-reagent, non-invasive and non-destructive analytical technique, which are great advantages if we consider that all current tests for detection of COVID-19 are cost- and time-consuming, require kits and reagents (mostly imported), and specialized human resources. If considering that in the near future its performance will be improved reaching as few false-negatives as possible, it is possible to suggest that this screening method could be applied at the entrance door to avoid unnecessary testing in patients with a 100% probability of being negative. Moreover, rapid identification of those suspect patients with high chance of infection by ATR-FTIR can influence decisions that need to be made before the RT-PCR results, such as the recommendation for quarantine or specific COVID units in case of hospitalization.

Our study has some limitations. It is worth noting that our study included relatively selected patient populations (i.e., individuals who sought a health service and were previously screened by a physician or nurse). Thus, we are aware of potential introduction of bias caused by not including patients with just one symptom or even asymptomatic, which could expand the applicability of ATR-FTIR to outside population studies. In addition, the results obtained in our study were limited by the fact that we did not use samples from patients also diagnosed with influenza. In a preliminary analysis, our results showed the effectiveness of the proposed diagnostic model FT-IR spectroscopy associated with machine learning of the differentiation of positive and negative COVID-19 patients. In future studies, we consider the possibility of including patient groups with different viral infections such as Influenza and H1N1 in order to corroborate our findings and consolidate the use of techniques in the medical and hospital environment.

Finally, we did not follow up for possible repeated RT-PCR on negative patients in the first testing. Because current findings indicate that RT-PCR test results from pharyngeal swab were variable and potentially unstable, and that initial RT-PCR tests may be negative and then become positive with repeated tests^35,36^, a patient that ATR-FTIR indicates as negative should not be considered as non-infected. However, we believe this is a proof-of-concept study demonstrating that ATR-FTIR, if combined with clinical, radiological and epidemiological criteria could be extremely useful as a real-time point-of-care strategy to reduce excessive and unnecessary expenses with RT-PCR in non-contaminated patients, or even to indicate early isolation and health care for patients with high probability to be COVID-19 positive cases, even before the final result by RT-PCR is available. In order to include ATR-FTIR analysis in the routine of medical facilities, a universal test should be developed with standardized instrumentation (with sufficient equipment specifications such as wavenumber resolution), standardized materials (such as collection swabs and VTM liquid for storage of the swab), standardized protocols for sample handling and storage, and a universal machine learning model for sample classification. A universal machine learning model will require increasing the number of patients as well as validating models using different combinations of instrumentation, materials and protocols. The most accurate model will determine the standardized combination to be used for ATR-FTIR testing. It is important to state that we are continuously increasing sample size in order to try subgrouping, subcategories, individualized algorithms and, thereby, enhance the performance (sensitivity and specificity) of FTIR in nasopharyngeal swab suspension fluid from COVID-19 patients.

In summary, such screening method by ATR-FTIR should be valuable in the current epidemic scenario, where the limitations of clinical and epidemiological diagnosis (similarity of the symptoms of COVID-19 infection with other high prevalent viruses) and the complexity and cost- and time-demanding of RT-PCR are critical problems for quick decision-making in emergency care. Furthermore, in regions with high prevalence, reduced testing of suspect cases should save RT-PCR test kits for patients with moderate and severe disease and for healthcare professionals (Supplementary Information).

## Conclusion

In the present proof-of-concept study, we concluded that FT-IR spectroscopy associated with artificial intelligence in nasopharyngeal swab suspension fluid was effective for discriminating between COVID-19 positive and negative patients and, in that way, our model can potentially be used for high-throughput screening for symptomatic suspect case.

## Methods

### Patient attendance and data collection

**Figure 11:**
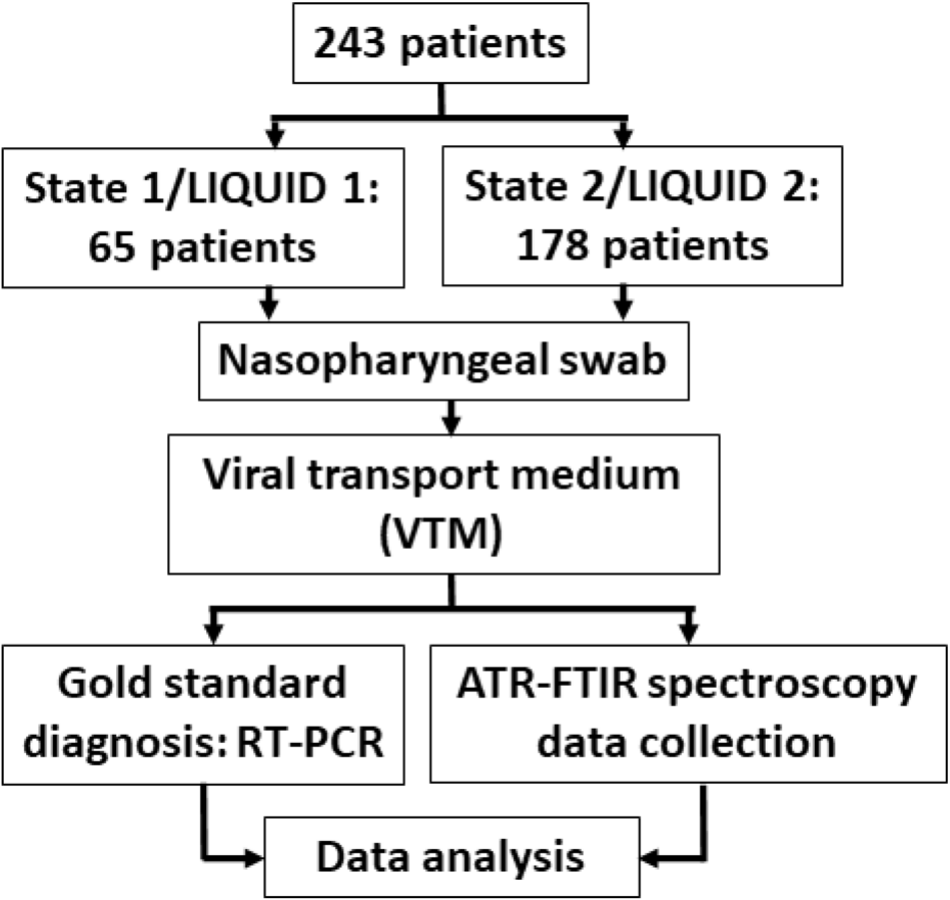
Data collection and analysis workflow.

The study was submitted to Plataforma Brasil and was evaluated by ethical committee of the Federal University of Espírito Santo. The study was approved under the numbers 30993920.1.0000.5071, 31411420.9.0000.8207 and 33838620.0.0000.5526. Informed consent was obtained for all patients participating in the study. All methods were carried out in accordance with relevant guidelines and regulations. Samples were obtained from individuals with more than 18 years old who RT-PCR were performed in three health care units in State 1 (Vila Velha Hospital, Hospital Roberto Arnizaut Silvares and Unidade Sanitária 3 in São Matheus, Espírito Santo, Brazil) and State 2 (Hospital Santa Casa de Misericórdia and Hospital de Base Luis Eduardo Magalhães, both in Itabuna, Bahia, Brazil) between May and July of 2020, and met the criteria for suspected cases according to State Health Secretary and World Health Organization (WHO) guidelines^37,38^. For all patients, clinical data (age, sex, pre-existing medical conditions, symptoms and date of onset of symptoms) were collected from medical records. A nasopharyngeal swab was collected from patients by inserting a swab into the nostril parallel to the palate. The swab was inserted to a location equidistant from the nostril and the outer opening of the ear and was gently scraped for a few seconds to absorb secretions. The rayon swab with a plastic shaft was placed immediately into a sterile tube containing 3 ml of the swab suspension fluid—viral transport medium (VTM). The same samples of these nasopharyngeal swab solutions employed for RT-PCR were used directly for FTIR analyses without sample preparation. All nasopharyngeal swabs were evaluated by RT-PCR that were performed in the central laboratory from the Health Secretary of each State (LACEN-SESA), as the gold standard method for definitive diagnosis of COVID-19 infection. It is important to point out that the liquids (i.e. the VTM compounds) used by these two Central Laboratories (State 1 and State 2) are different, and this was the reason why we did not combine the samples. The workflow of the present study is summarized in Fig. 11.

### ATR-FTIR measurements

Five microliters of each VTM (previously used for RT-PCR analysis) were air-dried on foil paper for 2 h and evaluated by a FTIR spectroscopy system (Cary 600 Agilent) coupled with an attenuated total reflection accessory. For samples collected in State 1 (LIQUID 1), we obtained 112 spectra from 40 COVID-19 positive patients and 74 spectra from 25 negative patients; and for those collected in State 2 (LIQUID 2), we measured 329 spectra from 111 COVID-19 positive patients, and 199 spectra from 67 negative patients. Samples were individually dried on 4 cm^2^ aluminum foils for 2 h inside the laminar flow, and afterwards, each set of aluminum foil + dry sample was placed on the ATR crystal in the FTIR. This procedure has been previously reported in the literature^39^ and was more feasible for the rapid processing and analysis of large quantities of samples and reduced the time for each measure in the ATR apparatus, because the samples are dried separately outside the crystal (i.e., on aluminum foils).

Although triplicate measurements were considered for each patient, some patients have different number of spectra collected due to outlier removal, in order to ensure the high quality of FTIR spectra used as an input to the classification model. Then, to generate the classification model in this study, each spectrum was considered an independent sample measurement to be subsequently included in each dataset. Wavenumbers of these spectra ranged from 650 cm^−1^ to 4000 cm^−1^, with a resolution of 1.86 cm^−1^.

### Data processing and statistical analysis

For the characterization of the sample and general and clinical data of the patients, a Kolmogorov–Smirnov normality test was performed, and the data are expressed as mean ± standard deviation. All steps of data pre-processing, machine learning and sample classification was performed by using the MATLAB (R2018a version, Mathworks, Natick, Massachusetts, United States) software.

For spectra pre-processing, the FTIR spectra of the viral transport medium (VTM) was subtracted from the raw FTIR spectra between 650 to 4000 cm^−1^ and the wavenumber range 650–3600 cm^−1^ was considered for analysis. Next, the FTIR spectra were smoothed by using a Savitsky–Golay filter (2nd polynomial order using 19 points). Then, we calculated the second derivative of the FTIR spectra. In this study, we reported the average FTIR spectra of COVID-19 positive and negative samples after the subtraction of the VTM spectra (Figs. 1A and 6A, Supplementary Information) and after computing the second derivative spectra (Figs. 1B and 6B). Average spectra were taken for each COVID-19 group and State separately. In order to avoid bias in feature selection for our classification model, we rescaled absorbance values at each wavenumber between − 1 and + 1. To develop our classification model, we used the Partial Least Squares (PLS) analysis associated with the K-Nearest Neighbours (KNN) classifier implemented in a MATLAB routine. Our model was built by using the second, third and fourth PLS components, which are correlated to the wavelengths shown in the loading plots of the PLS components (PLSCs). The first PLSC was excluded from the analysis due to the sample heterogeneity it featured, which could worsen the performance of the classifiers utilized in this study and would hinder the comparison between the datasets measured at the two locations where the data collection took place (Vitoria/State 1 and Itabuna/State 2). Therefore, PLSC1 could hinder the sample discrimination and was removed from the analysis. The PLS loadings of PLSC2, PLSC3 and PLSC4 were used to determine the main biochemical/structural components associated with this discrimination, while reducing the data dimensionality (fewer input parameters) for our classification model as well as the risk of overfitting. Biochemical components were determined based on the highest values of PLS loadings in the high wavenumber region between 2800–3000 cm^−1^ (which is not influenced by water absorption) and the fingerprint region between 650–1800 cm^−1^. One peak of the high wavenumber region and two of the fingerprint region were selected based on highest absolute values of PLS loadings and peak width smaller than the spectral resolution (4 cm^−1^) of the FTIR instrumentation.

In terms of the KNN classifier, we used 10 neighbours and calculations based on the cosine distance metric with no distance weight. In order to validate our classifiers, we first divided the datasets of Vitoria/State 1 and Itabuna/State 2 into two parts each. In terms of the Vitoria/State 1 dataset, we used the data collected from 40 positive patients (112 spectra) and 25 negative patients (74 spectra) to build the classification model. This model was validated by using k-fold cross-validation (when k = 5). In this type of validation, the dataset was randomly separated into training and tests sets with 80% and 20% of the total data, respectively. Next, the classification model was generated using the training set, and the model was applied to classify and validate the test set. Then, classification parameters such as sensitivity, specificity, accuracy and are under the receiver operating characteristic curve (AUC) were calculated as per definitions and equations below:

- True positive (TP): number of COVID-19 patients correctly classified
- False positive (FP): Healthy patients incorrectly classified
- True negative (TN): Healthy patients correctly classified
- False negative (FN): number of COVID-19 patients incorrectly classified
  1 $${\text{Sensitivity}} = \frac{{{\text{TP}}}}{{{\text{TP}} + {\text{FN}}}},$$
  2 $${\text{Specificity}} = \frac{{{\text{TN}}}}{{{\text{TN}} + {\text{FP}}}},$$
  3 $${\text{Accuracy}} = \frac{{{\text{TP}} + {\text{TN}}}}{{{\text{TP}} + {\text{FN}} + {\text{TN}} + {\text{FP}}}}.$$

**Figure 12:**
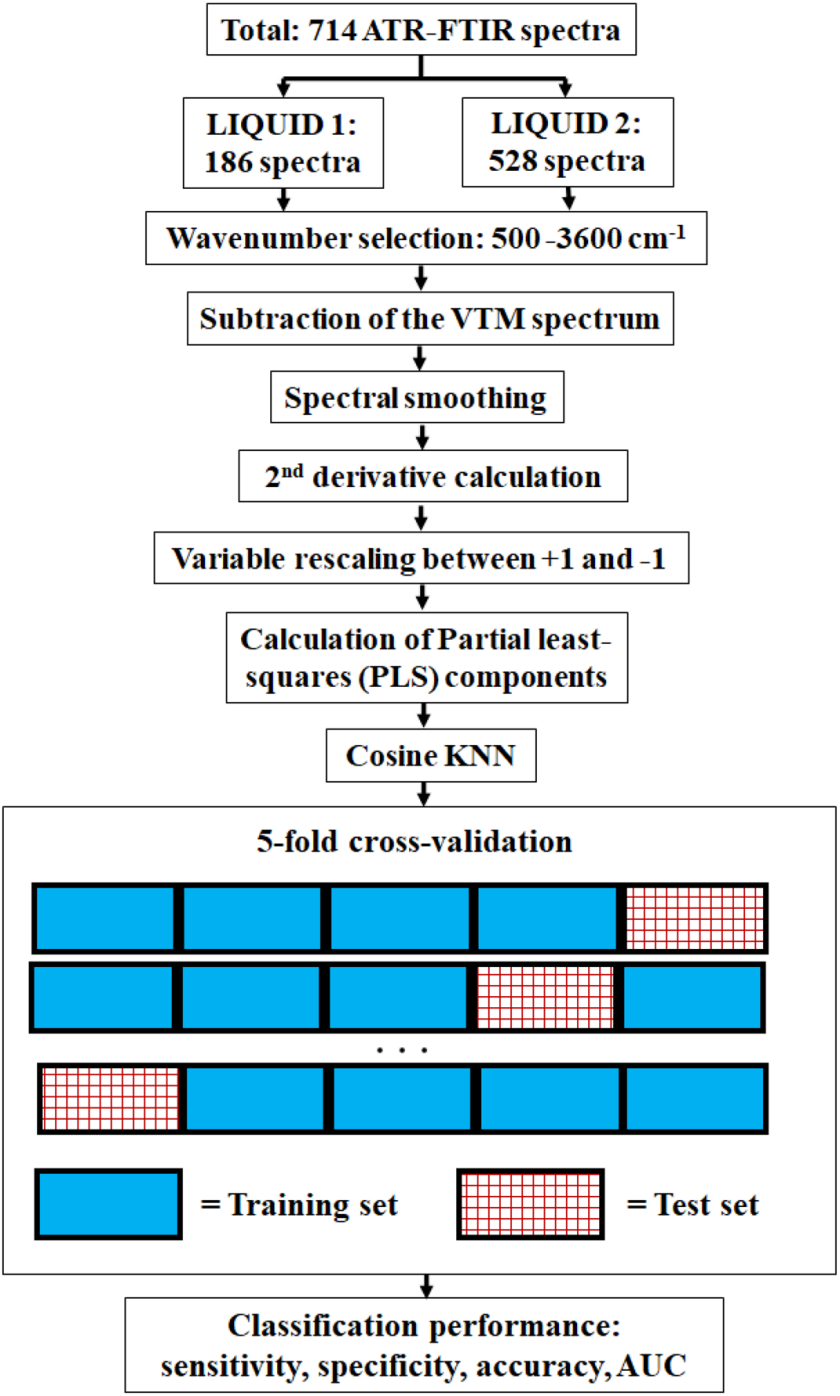
Steps of data analysis of the ATR-FTIR spectra from COVID-19 VTM LIQUID samples.

The process was repeated five times until all parts of the datasets were included in the test set. The mean of the classification performance parameters was calculated and reported. The entire same process was repeated for the Itabuna/State 2 dataset, which consisted of FTIR spectra from 111 positive patients (329 spectra) and 67 negative patients (199 spectra) to build the classification model. A summary of the data analysis workflow is shown in Fig. 12.

## Supplementary Information

Supplementary Figures.

## References

[1] Loeffelholz MJ, Tang Y-W (2020). Laboratory diagnosis of emerging human coronavirus infections—The state of the art. Emerg. Microbes Infect..

[2] Peng X (2020). Transmission routes of 2019-nCoV and controls in dental practice. Int. J. Oral Sci..

[3] World Health Organization (WHO). Pneumonia of unknown cause—China. https://www.who.int/csr/don/05-january-2020-pneumonia-of-unkown-cause-china/en/ (2020). Accessed 13 Dec 2020

[4] World Health Organization (WHO). Coronavirus disease 2019 (COVID-19)—Situation Report 51. (2020).

[5] Saito Nogueira, M. Biophotonics for pandemic control: Large-area infection monitoring and microbial inactivation of COVID-19. *Photodiagn. Photodyn. Ther.* **31**, 101823 (2020). 10.1016/j.pdpdt.2020.101823 PMC7238977 32445960

[6] Nogueira, M. S. Optical theranostics and treatment dosimetry for COVID-19 lung complications: Towards increasing the survival rate of vulnerable populations. *Photodiagn. Photodyn. Ther.* **31**, 101892 (2020). 10.1016/j.pdpdt.2020.101892 PMC7306734 32585401

[7] Nogueira, M. S. Ultraviolet-based biophotonic technologies for control and prevention of COVID-19, SARS and related disorders. *Photodiagn. Photodyn. Ther.* **31**, 101890 (2020). 10.1016/j.pdpdt.2020.101890 PMC7308021 32585400

[8] Udugama B (2020). Diagnosing COVID-19: The disease and tools for detection. ACS Nano.

[9] Böger, B. *et al.* Systematic review with meta-analysis of the accuracy of diagnostic tests for COVID-19. *Am. J. Infect. Control* **49(1)**, 21–29 (2020). 10.1016/j.ajic.2020.07.011 PMC7350782 32659413

[10] Sethuraman N, Jeremiah SS, Ryo A (2020). Interpreting diagnostic tests for SARS-CoV-2. JAMA.

[11] Ren, X. *et al.* Application and Optimization of RT-PCR in Diagnosis of SARS-CoV-2 Infection. *Preprints with The Lancet,* (2020).

[12] Carvalho LFCS, Nogueira MS, Neto LPM, Bhattacharjee TT, Martin AA (2017). Raman spectral post-processing for oral tissue discrimination—A step for an automatized diagnostic system: Erratum. Biomed. Opt. Express.

[13] Carvalho LFCS, Nogueira MS, Neto LPM, Bhattacharjee TT, Martin AA (2018). Raman spectral post-processing for oral tissue discrimination—A step for an automatized diagnostic system: erratum. Biomed. Opt. Express.

[14] Carvalho LFCS (2019). In vivo Raman spectroscopic characteristics of different sites of the oral mucosa in healthy volunteers. Clin. Oral Investig..

[15] Leal LB, Nogueira MS, Canevari RA, Carvalho LFCS (2018). Vibration spectroscopy and body biofluids: Literature review for clinical applications. Photodiagn. Photodyn. Ther..

[16] das e Silva LFC, Nogueira MS (2018). New insights of Raman spectroscopy for oral clinical applications. Analyst.

[17] Baker MJ (2018). Clinical applications of infrared and Raman spectroscopy: State of play and future challenges. Analyst.

[18] Pizarro C, Esteban-Diez I, Arenzana-Rámila I, González-Sáiz JM (2018). Discrimination of patients with different serological evolution of HIV and co-infection with HCV using metabolic fingerprinting based on Fourier transform infrared. J. Biophotonics.

[19] Carvalho, L. F. C. S. & Saito Nogueira, M. Optical techniques for fast screening—Towards prevention of the coronavirus COVID-19 outbreak. *Photodiagn. Photodyn. Ther.* **30**, 101765 (2020). 10.1016/j.pdpdt.2020.101765 PMC7158832 32304912

[20] Nogueira, M. S. Biophotonic telemedicine for disease diagnosis and monitoring during pandemics: Overcoming COVID-19 and shaping the future of healthcare. *Photodiagn. Photodyn. Ther.* **31**, 101836 (2020). 10.1016/j.pdpdt.2020.101836 PMC7255238 32473399

[21] Movasaghi Z, Rehman S, ur Rehman DI (2008). Fourier transform infrared (FTIR) spectroscopy of biological tissues. Appl. Spectrosc. Rev..

[22] Naseer K, Ali S, Qazi J (2021). ATR-FTIR spectroscopy as the future of diagnostics: A systematic review of the approach using bio-fluids. Appl. Spectrosc. Rev..

[23] Sharma O, Sultan AA, Ding H, Triggle CR (2020). A review of the progress and challenges of developing a vaccine for COVID-19. Front. Immunol..

[24] Chan JF-W (2020). Improved molecular diagnosis of COVID-19 by the novel, highly sensitive and specific COVID-19-RdRp/Hel real-time reverse transcription-PCR assay validated in vitro and with clinical specimens. J. Clin. Microbiol..

[25] Chakraborty C, Sharma AR, Sharma G, Bhattacharya M, Lee SS (2020). SARS-CoV-2 causing pneumonia-associated respiratory disorder (COVID-19): Diagnostic and proposed therapeutic options. Eur. Rev. Med. Pharmacol. Sci..

[26] Gurbanov R, Tunçer S, Mingu S, Severcan F, Gozen AG (2019). Methylation, sugar puckering and Z-form status of DNA from a heavy metal-acclimated freshwater *Gordonia* sp.. J. Photochem. Photobiol. B Biol..

[27] Gurbanov RS, Ozek N, Tunçer S, Severcan F, Gozen AG (2018). Aspects of silver tolerance in bacteria: Infrared spectral changes and epigenetic clues. J. Biophotonics.

[28] Butler HJ (2019). Development of high-throughput ATR-FTIR technology for rapid triage of brain cancer. Nat. Commun..

[29] Carvalho LFCS (2015). Raman micro-spectroscopy for rapid screening of oral squamous cell carcinoma. Exp. Mol. Pathol..

[30] Carvalho LFCS (2017). Raman spectroscopic analysis of oral cells in the high wavenumber region. Exp. Mol. Pathol..

[31] das Carvalho LFCS (2016). Optical diagnosis of actinic cheilitis by infrared spectroscopy. Photodiagn. Photodyn. Ther..

[32] Roman M, Wrobel TP, Paluszkiewicz C, Kwiatek WM (2020). Comparison between high definition FT-IR, Raman and AFM-IR for subcellular chemical imaging of cholesteryl esters in prostate cancer cells. J. Biophotonics.

[33] Zhu, N. *et al.* A novel coronavirus from patients with pneumonia in China, 2019. *N. Engl. J. Med.* **382**, 727–733 (2020). 10.1056/NEJMoa2001017 PMC7092803 31978945

[34] Ye Z, Zhang Y, Wang Y, Huang Z, Song B (2020). Chest CT manifestations of new coronavirus disease 2019 (COVID-19): A pictorial review. Eur. Radiol..

[35] Zitek T (2020). The appropriate use of testing for COVID-19. West. J. Emerg. Med..

[36] Li, Y. *et al.* Stability issues of RT-PCR testing of SARS-CoV-2 for hospitalized patients clinically diagnosed with COVID-19. *J. Med. Virol.* **92(7)**, 903–908 (2020). 10.1002/jmv.25786 PMC7228231 32219885

[37] Secretaria da Saude & Governo do Estado do Espirito Santo. *Technical note COVID-19 N° 53/2020-GEVS/SESA/ES*.

[38] World Health Organization (WHO). Technical guidance publications - https://www.who.int/emergencies/diseases/novel-coronavirus-2019/technical-guidance-publications. Accessed 13 Dec 2020

[39] Paraskevaidi M (2018). Aluminium foil as an alternative substrate for the spectroscopic interrogation of endometrial cancer. J. Biophotonics.

