# Author Correction: Rapid diagnosis of COVID-19 using FT-IR ATR spectroscopy and machine learning

**DOI:** 10.1038/s41598-021-02660-x

**Published:** 2021-11-24

**Authors:** Marcelo Saito Nogueira, Leonardo Barbosa Leal, Wena Dantas Marcarini, Raquel Lemos Pimentel, Matheus Muller, Paula Frizera Vassallo, Luciene Cristina Gastalho Campos, Leonardo dos Santos, Wilson Barros Luiz, José Geraldo Mill, Valerio Garrone Barauna, Luis Felipe das Chagas e Silva de Carvalho

**Affiliations:** 1 Tyndall National Institute, University College Cork, Lee Maltings Complex, Dyke Parade, Cork, T12R5CP Ireland; 2 Department of Physiological Sciences, Federal University of Espírito Santo (UFES), Vitória, Brazil; 3 Universidade de Taubaté, Taubaté, Brazil; 4 Centro Universitário Braz Cubas, Mogi das Cruzes, Brazil; 5 Clinical Hospital, Federal University of Minas Gerais, Belo Horizonte, MG Brazil; 6 Department of Biological Science, Santa Cruz State University, Ilhéus, BA Brazil; 7 Faculdade Vale do Cricaré, São Matheus, Brazil

Correction to: *Scientifc Reports* https://doi.org/10.1038/s41598-021-93511-2, published online 11 October 2021

The original version of this Article contained an error in the spelling of the author Wena Dantas Marcarini which was incorrectly given as Wena Macarini. The original Article has been corrected.

