# Supplementary material for: Rapid diagnosis of COVID-19 using FT-IR ATR spectroscopy and machine learning
Source: Sci Rep. 2021 Oct 11;11:15409. doi: 10.1038/s41598-021-93511-2 (PMC8505540; doi:10.1038/s41598-021-93511-2)
Supplement: Supplementary file 1 — Supplementary Figures. [file 41598_2021_93511_MOESM1_ESM.pdf]

Supplementary material

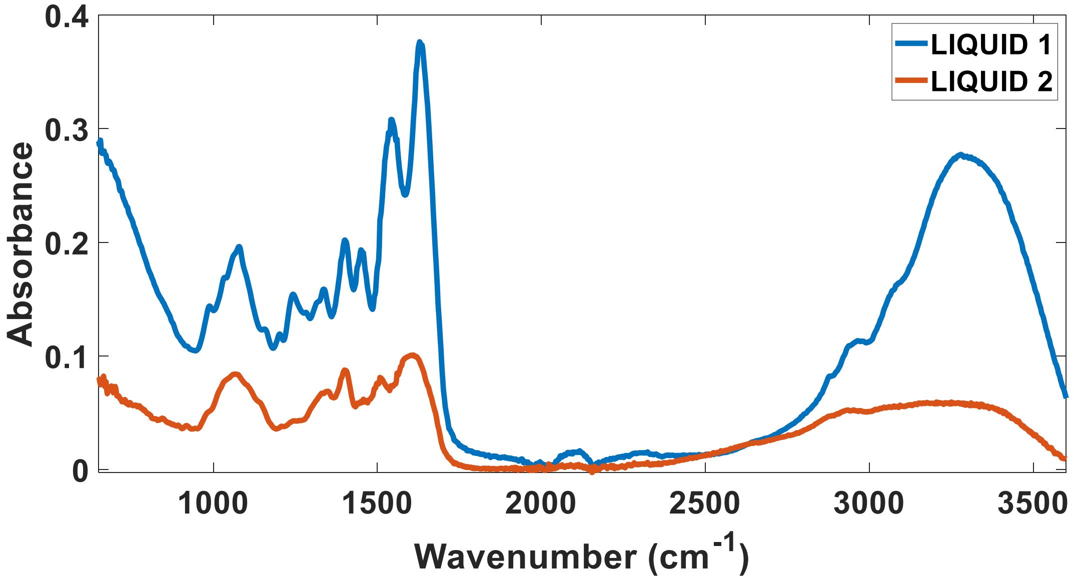

Figure 1: FTIR spectrum of the viral transport medium (VTM) of State 1 (LIQUID 1) and that of State 2 (LIQUID 2).
